# Mental health of individuals with pre-existing mental illnesses at the beginning of the COVID-19 pandemic: results of the German National Cohort (NAKO)

**DOI:** 10.3389/fpubh.2024.1451631

**Published:** 2024-09-23

**Authors:** Janine Stein, Alexander Pabst, Klaus Berger, André Karch, Henning Teismann, Fabian Streit, Hans J. Grabe, Rafael Mikolajczyk, Janka Massag, Wolfgang Lieb, Stefanie Castell, Jana-Kristin Heise, Matthias B. Schulze, Sylvia Gastell, Volker Harth, Nadia Obi, Annette Peters, Marie-Theres Huemer, Patricia Bohmann, Michael Leitzmann, Sabine Schipf, Claudia Meinke-Franze, Antje Hebestreit, Daniela C. Fuhr, Karin B. Michels, Stefanie Jaskulski, Hannah Stocker, Lena Koch-Gallenkamp, Stefan N. Willich, Thomas Keil, Markus Löffler, Kerstin Wirkner, Steffi G. Riedel-Heller, for German National Cohort (NAKO) Consortium

**Affiliations:** ^1^Institute of Social Medicine, Occupational Health and Public Health (ISAP), University of Leipzig, Leipzig, Germany; ^2^Institute of Epidemiology and Social Medicine, University of Münster, Münster, Germany; ^3^Department of Genetic Epidemiology in Psychiatry, Medical Faculty Mannheim, Central Institute of Mental Health, University of Heidelberg, Heidelberg, Germany; ^4^Department of Psychiatry and Psychotherapy, University Medicine Greifswald, Greifswald, Germany; ^5^Interdisciplinary Centre for Health Sciences, Institute for Medical Epidemiology, Biometrics and Informatics (IMEBI), Medical Faculty of the Martin Luther University Halle-Wittenberg, Halle (Saale), Germany; ^6^German Center for Mental Health (DZPG), partner site Halle-Jena-Magdeburg, Magdeburg, Germany; ^7^Institute of Epidemiology, Faculty of Medicine, Kiel University, Kiel, Germany; ^8^Department of Epidemiology, Helmholtz Centre for Infection Research, Brunswick, Germany; ^9^Department of Molecular Epidemiology, German Institute of Human Nutrition Potsdam-Rehbruecke, Nuthetal, Germany; ^10^Institute of Nutritional Science, University of Potsdam, Nuthetal, Germany; ^11^NAKO Study Center, German Institute of Human Nutrition Potsdam-Rehbruecke, Nuthetal, Germany; ^12^Institute of Occupational and Maritime Medicine (ZfAM), University Medical Center Hamburg-Eppendorf, Hamburg, Germany; ^13^Institute of Epidemiology, Helmholtz Zentrum München - German Research Center for Environmental Health (GmbH), Neuherberg, Germany; ^14^Chair of Epidemiology, Medical Faculty, Institute for Medical Information Processing, Biometry and Epidemiology, Ludwig-Maximilians-Universität München, Munich, Germany; ^15^German Center for Mental Health (DZPG), partner site München-Augsburg, Augsburg, Germany; ^16^Department of Epidemiology and Preventive Medicine, University of Regensburg, Regensburg, Germany; ^17^Institute for Community Medicine, Department SHIP/Clinical-Epidemiological Research, University Medicine Greifswald, Greifswald, Germany; ^18^Leibniz Institute for Prevention Research and Epidemiology – BIPS, Bremen, Germany; ^19^Health Sciences, University of Bremen, Bremen, Germany; ^20^Faculty of Medicine and Medical Center, Institute for Prevention and Cancer Epidemiology, University of Freiburg, Freiburg, Germany; ^21^Division of Clinical Epidemiology and Aging Research, German Cancer Research Center (DKFZ), Heidelberg, Germany; ^22^Institute of Social Medicine, Epidemiology and Health Economics, Charité - Universitätsmedizin Berlin, Berlin, Germany; ^23^Institute of Clinical Epidemiology and Biometry, University of Würzburg, Würzburg, Germany; ^24^State Institute of Health I, Bavarian Health and Food Safety Authority, Erlangen, Germany; ^25^Institute for Medical Informatics, Statistics, and Epidemiology, University of Leipzig, Leipzig, Germany; ^26^Leipzig Research Center for Civilization Diseases LIFE, Medical Faculty, University of Leipzig, Leipzig, Germany

**Keywords:** COVID-19 pandemic, mental health, depression, anxiety, longitudinal cohort study, German National Cohort (NAKO)

## Abstract

**Background:**

The COVID-19 pandemic prompted a range of studies on mental health, with mixed results. While numerous studies reported worsened conditions in individuals with pre-existing mental disorders, others showed resilience and stability in mental health. However, longitudinal data focusing on the German population are sparse, especially regarding effects of age and pre-existing mental disorders during the early stages of the pandemic.

**Objectives:**

To assess the interplay between psychiatric history, age, and the timing of the pandemic, with a focus on understanding how these factors relate to the severity of depression and anxiety symptoms.

**Methods:**

Exploratory analyses were based on 135,445 individuals aged 20–72 years from the German National Cohort (NAKO). Depressive and anxiety symptoms were assessed before and after the first wave of the pandemic. Inferential statistical analyses and negative binomial regression models were calculated.

**Results:**

Persons with a self-reported psychiatric history exhibited comparable levels of depression and anxiety symptom severity after the first wave of the pandemic compared to the time before. In contrast, individuals without a psychiatric history, particularly those in their 20s to 40s, experienced an increase in mental health symptom severity during the first wave of the pandemic.

**Limitations:**

Analyses focuses on the first wave of the pandemic, leaving the long-term mental health effects unexplored.

**Conclusion:**

Future research should consider age-specific and mental-health-related factors when addressing global health crises. Additionally, it is important to explore factors influencing resilience and adaptation, aiming to develop targeted interventions and informed policies for effective mental health management during pandemics.

## Introduction

Persistently growing research on the psychological consequences of the SARS-CoV-2 pandemic focused on several groups of individuals including the general population, medical staff, directly infected persons and other patient groups (1). In this context, people with pre-existing mental health problems represent a special group of interest, as they seem to be particularly vulnerable to adverse effects of the COVID-19 pandemic (2). Up to date, numerous studies have been published since the beginning of the pandemic. Recent findings regarding people with pre-existing mental illness were contradictory. On the one hand, immediate mental reactions of people with pre-existing mental illnesses comprised adverse effects on psychopathology (e.g., intensification of pre-existing psychological symptoms). On the other hand, findings suggested unexpected resilience of patients with pre-existing mental health problems and observed that psychiatric symptoms remained stable across the first months of the pandemic or even slightly decreased, partly depending on underlying diagnoses (3). A recent narrative systematic review assessed 97 studies covering several symptom clusters including depression and anxiety. Overall, the authors concluded that pre-existing mental health diagnoses were not associated with an exacerbation of symptoms. However, findings for depression in psychiatric groups did not follow a clear pattern. While many studies found an increase in depression severity, other studies reported no change or even a decrease in symptom severity over the course of the pandemic. Reported effect sizes were mostly small. Further, anxiety seemed to be an exception and psychiatric samples tended to show an increase in anxiety. Since there are only a few studies reporting on anxiety, and most of the studies that reported null results were based on unselected samples, small sample sizes or had small statistical power, more research is needed to understand the mechanisms (4). Despite the high volume of already existing studies in this field, there is still a high demand for longitudinal data (pre-and post-pandemic). Analyses should be based on the usage of established instruments and scales to assess psychiatric conditions during reactions to the outbreak as well as the short-and long-term psychological consequences under health protection actions (lockdown, social distancing, and restricted supply of mental health services).

To date, a large body of studies published in the first year of the pandemic focused on individuals with pre-existing mood disorders including depression, anxiety, and specific stress-related disorders (5). In the German-speaking countries, studies that have examined the consequences of the pandemic in the group of people with pre-existing mental health problems on a longitudinal basis are rare. Previous studies were mostly cross-sectional and with a small sample size (6–8). Additionally, recent review and meta-analytic data indicated a heterogeneous quality of studies, low certainty of evidence in all outcomes due to risk of bias, inconsistency, indirectness, and imprecision of measures (3, 9). Furthermore, little is known about how the pandemic has affected the level or severity of symptoms in people with pre-existing psychiatric conditions compared to people without a pre-existing condition. Age may also play a crucial role since systematic analyses showed that older compared to younger individuals were at particular risk for the pandemic’s negative mental health consequences (10). However, findings regarding age were contradictory (1).

Against this background, the current exploratory study aims to examine whether people with compared to people without a psychiatric history experienced a stronger psychological reaction to the outbreak and the health protection measures during the COVID-19 pandemic (first lockdown, social distancing) compared to people without such a history. Specifically, the study intends to answer the question whether people who have previously suffered from a psychiatric disorder react with increased psychiatric burden to the outbreak and the health protection measures. In our analyses, psychiatric history focused on depression and anxiety and we further investigated the age of the participants as a potential influencing factor. In extension to previous research, analyses of this study are based on the original data of a large longitudinal population-based German sample of adults—the German National Cohort (NAKO)—covering a wide range of the lifespan, enabling the assessment of age-related levels of improvement or deterioration in mental health outcomes. Specifically, we aim to examine the associations between psychiatric history, age and time before/during the pandemic with symptom severity of depression and anxiety. Moreover, we intend to explore the interactions and dependencies between psychiatric history, age and time before/during the pandemic in relation to the symptom severity.

## Methods

### Study design

Data for this study was derived from the German National Cohort (NAKO), a population-based cohort study examining 205,415 randomly selected individuals aged 20–72 years across 18 study centers and 16 regions of Germany (11–13). Baseline examinations were carried out between 2014 and 2019 comprising two levels. At Level-1 examinations (L1; 3–4 h), all participants were assessed in the study centers following a standardized protocol. Level-2 examinations were more detailed and conducted in a subset of those participants who volunteered to participate again (20%). The present study also includes the data of a first COVID-19 pandemic-focused special survey – that took place from May to November 2020 and comprised approximately 81.8% of the original baseline sample. Consequently, the current analyses are based on the comparison of data before the pandemic (baseline) and during the first wave of the pandemic (follow-up). Detailed information on the NAKO and the used database can be found elsewhere (12, 14–16). The study has received ethics committee approval of all participating centers.

### Study sample

The initial sample at baseline comprised 204.867 participants. For this study, participants were excluded from the initial sample if they did not take part at the COVID-19 follow-up survey (*n* = 42,952), if they had invalid or missing test scores on the 9-item Patient Health Questionnaire (PHQ-9) measuring depression (*n* = 16,850) and/or the 7-item Generalized Anxiety Disorder Scale (GAD-7) measuring anxiety (*n* = 3,156), and if they had missing values for the life-time diagnoses of depression/anxiety (*n* = 1,119), or the selected covariates (*n* = 5,345). Finally, the analyses were based on a sample of *n* = 135,445 individuals aged 20–72 years [51.15% female, mean (SD) age = 50.23 (12.09) years].

### Instruments and procedures

Mental health of participants was operationalized via depressive and anxiety symptoms, assessed using the following standardized instruments both at baseline and the first COVID-19 follow-up survey.

### PHQ-9

Participants were assessed via digitalized self-report questionnaires including the German version of the PHQ-9 (17) for the dimensional assessment of depressive symptoms. The PHQ-9 was filled out by participants on a touch screen. Following the symptoms comprising Criterion A in DSM-IV, the PHQ-9 was developed for the screening of depression. On a scale from 0 (“not at all”) to 3 (“almost every day”) participants were asked about the presence of symptoms in the last 2 weeks. The sum score ranges from 0 to 27 with higher values indicating more severe depressive symptoms. Score ranges were suggested to grade the severity of depression: 0–4 minimal, 5–9 mild, 10–14 moderate, 15–19 moderately severe, and 20–27 severe. To indicate the presence of a current depressive episode, the cut-off score ≥ 10 (moderate to severe symptoms) was used (17, 18).

### GAD-7

Anxiety symptoms and severity in the present analysis were assessed via the German version of the GAD-7 (19). In the present cohort, a cutoff of GAD-7 ≥ 8 was used to screen for anxiety disorder (AD) and of GAD-7 ≥ 10 to identify clinically meaningful symptom severity level indicating generalized anxiety disorder (GAD). On a scale ranging from 0 (“not at all”) to 3 (“nearly every day”) participants were asked how often they have been bothered by symptoms of anxiety, for example, feeling nervous and anxious, being worried and restless. In the NAKO, seven core symptoms of AD and GAD according to DSM-criteria over the last 4 weeks were depicted via the GAD-7 scale (20). The sum score ranges from 0 to 21 with higher values representing a higher severity level of anxiety symptoms. Severity levels of anxiety are defined as scores ranging from 0–4: minimal anxiety, 5–9: mild anxiety, 10–14: moderate anxiety, 15–21: severe anxiety. For the GAD-7, substantial psychometric properties in terms of reliability and validity (criterion, construct, factorial, and procedural) were shown (21).

### Psychiatric history (self-report)

To determine whether psychiatric illness had ever occurred, participants were asked at the baseline assessment whether a doctor or psychotherapist had ever diagnosed them with depression. The same question was asked regarding anxiety disorders.

### Other variables

Since time between baseline and follow-up ranged between 0.6 and 6.7 years (mean 3.2, SD 1.2 years) across individuals, age was assessed at both time points. In addition, time was dummy-coded in the analyses, differentiating between baseline and follow-up assessment. Further, baseline information for sociodemographic variables including gender, migration background, marital status, educational attainment, profession/occupational, and current employment status were included in the models to account for possible confounding effects. For retired participants, the predominant occupation group during working life was considered.

### Statistical analyses

All analyses were performed using Stata version 17.0 BE (Stata Corp LP, College Station, TX). Descriptive statistics were calculated as mean with standard deviation (SD) or absolute frequencies and percentages. Gender comparisons were tested using *t*-tests (continuous) or Pearson chi-square tests (categorial), as appropriate. Comparisons of depression and anxiety measures over time (baseline vs. follow-up) were evaluated using either paired *t*-tests or McNemar tests for matched pairs.

Associations of age, time and psychiatric history with severity of depression and anxiety were estimated using negative binomial regression models, with mental disorder severity operationalized as symptom count. This was considered the best GLM specification, as based on graphical inspection (overdispersed data for PHQ-9 and GAD-7 scores), and further affirmed by the modified Park test. Specifically, we used multilevel mixed-effects generalized linear models (GLMs) with negative binomial distribution and log-link function to account for the panel structure of the data by including a random intercept for participants. A series of main and interaction effects regression analyses for modeling both depression and anxiety severity were conducted. Main effects models included age (at baseline and follow-up, respectively), time (baseline vs. follow-up) and psychiatric history (yes vs. no) in order to examine independent effects on mental disorder severity. Since we assumed nonlinear relationships of age with both depression and anxiety severity (22), age was included as restricted cubic splines with 7 knots at fixed centiles (23). The interaction models for depression and anxiety additionally included 2-way interaction terms for age X time, age X psychiatric history and time X psychiatric history, and a 3-way interaction age X time X psychiatric history to model interdependencies of these risk factors. All models were adjusted for gender, migration background, marital status, educational attainment, profession, and current employment status.

Results of the regression models are reported as incidence rate ratios (IRR) with 95% confidence intervals, which can be re-expressed as percentage change in the number of symptoms associated with a 1-unit increase in the predictor (24). Wald tests are reported to evaluate the significance of qualitative main and interaction effects. Estimates for the association of nonlinear age with outcomes at representative values of age were calculated using the *xblc* algorithm in Stata and are presented as IRRs with age 45 years as reference (25). Graphs of point and interval estimates for predictions of the mean number of depression and anxiety symptoms evaluated at 2-year increments of age were computed using the *postrcspline* module in Stata and are presented for both time points and stratified for psychiatric history (26). The design of the NAKO was considered in all analyses by correcting the variance estimators for clustering by study center.

## Results

### Sample characteristics

Table 1 illustrates the sociodemographic characteristics of the patient sample at baseline. Mean age of participants at baseline was 50.23 years (SD = 12.09), almost 51% were female. At follow-up, mean age was 53.40 (SD = 12.38). Most of the participants (60.80%) were married. Almost half of the sample had an advanced technical college degree or baccalaureate (55.90%) and most participants were employed (86.99%) in a full time position (53.57%). In the total sample, the mean PHQ-9 score was 3.70 and the mean GAD-7 score was 3.03 at baseline. Except for migration background, differences were found between women and men across all sociodemographic characteristics as well as mean depression and anxiety scores. Of the 135,445 participants of the total sample, 18,695 (13.80%) participants reported a lifetime diagnosis of depression. Of these participants, 12,132 (64.89%) were female and 6,563 (35.11%) were male. Further, 9,835 (7.26%) of the total sample reported a lifetime diagnosis of anxiety. Of these participants, 6,463 (65.44%) were female and 3,399 (34.56%) were male. There was some variation in the observed frequencies of the assessed psychiatric history of depression and anxiety (i.e., lifetime diagnosis depression and anxiety) between the study centers. The frequency of a physician’s diagnosis of depression ranged from12.4% (Neubrandenburg) to 18.6% (Berlin South), and of anxiety from 3.9% (Münster) to 6.6% (Essen) (14, 20).

**Table 1:** Sociodemographic and clinical characteristics of the sample.

		Participants		
	Total sample (*N* = 135,445)	Female (*n* = 69,279, 51.15%)	Male (*n* = 66,166, 48.85%)	*p*-value^a^ (test statistic)
**Age (in years) at baseline**				
*M* (SD)	50.23 (12.09)	49.88 (12.08)	50.59 (12.10)	<0.001 (*t* = −10.733)
Range	20–72	20–72	20–72	
**Age (in years) at follow-up**				
*M* (SD)	53.40 (12.38)	53.13 (12.33)	53.68 (12.43)	<0.001 (*t* = −8.142)
Range	20–76	21–76	20–76	
**Migration background [*n*, (%)]**				
Yes	40,327 (29.77)	20,550 (29.66)	19,777 (29.89)	0.36 (*Χ*^2^ = 0.836)
No	95,118 (70.23)	48,729 (70.34)	46,389 (70.11)	
**Marital status [*n*, (%)]**				
Married/living with spouse	82,344 (60.80)	40,108 (57.89)	42,236 (63.83)	<0.001 (*Χ*^2^ = 1.7e+03)
Married/living apart	2,231 (1.65)	1,213 (1.75)	1,018 (1.54)	
Single/unmarried living alone or with partner	33,800 (24.95)	16,964 (24.49)	16,836 (25.45)	
Divorced	13,591 (10.03)	8,284 (11.96)	5,307 (8.02)	
Widowed	3,479 (2.57)	2,710 (3.91)	766 (1.16)	
**Educational attainment [*n*, (%)]**				
No degree	1,678 (1.24)	784 (1.13)	894 (1.35)	<0.001 (*Χ*^2^ = 1.1e+03)
General elementary school	16,146 (11.92)	7,261 (10.48)	8,885 (13.43)	
Secondary/polytechnic school	41,902 (30.94)	24,076 (34.75)	17,826 (26.94)	
Advanced technical college/baccalaureate	75,719 (55.90)	37,158 (53.64)	38,561 (58.28)	
**Profession [*n*, (%)]**				
Employee	117,828 (86.99)	62,213 (89.80)	55,615 (84.05)	<0.001 (*Χ*^2^ = 1.2e+03)
Self-employed	16,575 (12.24)	6,438 (9.29)	10,137 (15.32)	
Other (e.g., apprenticeship)	1,042 (0.77)	628 (0.91)	414 (0.63)	
**Current employment [*n*, (%)]**				
Full-time	72,560 (53.57)	27,084 (39.09)	45,476 (68.73)	<0.001 (*Χ*^2^ = 1.6e+04)
Part-time	32,262 (23.82)	25,465 (36.76)	6,797 (10.27)	
Unemployed	3,858 (2.85)	1,748 (2.52)	2,110 (3.19)	
Non-employee	26,765 (19.76)	14,982 (21.63)	11,783 (17.81)	
**PHQ-9**				
*M* (SD)	3.70 (3.53)	4.15 (3.67)	3.23 (3.31)	<0.001 (*t* = 48.611)
Cutoff ≥ 10 (*n*, %)	8,927 (6.59)	5,603 (8.09)	3,324 (5.02)	<0.001 (*Χ*^2^ = 516.035)
**GAD-7**				
M (SD)	3.03 (3.09)	3.47 (3.27)	2.58 (2.83)	<0.001 (*t* = 52.964)
Cutoff ≥ 10 (*n*, %)	6,020 (4.44)	3,945 (5.69)	2,075 (3.14)	<0.001 (*Χ*^2^ = 521.546)
**Lifetime diagnosis depression**	18,695 (13.80)	12,132 (17.51)	6,563 (9.92)	<0.001 (*Χ*^2^ = 1.6e+03)
**Lifetime diagnosis anxiety**	9,835 (7.26)	6,436 (9.29)	3,399 (5.14)	<0.001 (*Χ*^2^ = 866.767)

M = mean; SD = standard deviation; ^a^comparison of female and male participants were based on Pearson chi-square-tests (Χ²) or *t*-tests, as appropriate; PHQ-9 = 9-item Patient Health Questionnaire; GAD-7 = 7-item Generalized Anxiety Disorder Scale.

### Depression and anxiety in participants with or without self-reported psychiatric history

Table 2 shows the mean scores at baseline (before the pandemic) and follow-up (during first wave of the pandemic) assessments for depression (PHQ-9) and anxiety (GAD-7) depending on the self-reported lifetime psychiatric diagnoses. Comparisons between groups revealed differences between participants with or without a lifetime psychiatric diagnosis: participants with a lifetime diagnosis of depression or anxiety reported mean scores almost twice as high for depression and anxiety at both time points compared to participants without a psychiatric history in their lives. Considering evaluated cut-offs, 24.72% of the participants with a psychiatric history were classified as depressed at baseline and 23.31% at follow-up. Regarding anxiety, 19.76% of the participants showed clinically relevant anxiety symptoms at baseline and 16.76% at follow-up. In participants with a psychiatric history, the trend showed a decrease in symptom severity after the first wave of the pandemic compared with the time point before the pandemic for both depression and anxiety. In contrast, participants without a psychiatric history initially showed an increase in depression and anxiety symptomatology after the first wave of the pandemic compared with before the pandemic.

**Table 2:** Characteristics of depression and anxiety at baseline and follow-up assessments depending on self-reported psychiatric history of depression or anxiety disorder, respectively.

	No psychiatric history			Psychiatric history		
	BL	FU	*p*-value^a^ (test statistic)	BL	FU	*p*-value^a^ (test statistic)
**Depression: PHQ-9**						
*M* (*n*; SD)	3.21 (116,750; 2.92)	3.60 (116,750; 3.52)	<0.001 (z = −31.871)	6.80 (186,695; 5.09)	6.53 (186,695; 5.16)	<0.001 (*z* = 7.483)
Cutoff ≥ 10 [*n*, (%)]	4,305 (3.69)	7,626 (6.53)	<0.001 (*Χ*^2^ = 1245.52)	4,622 (24.72)	4,357 (23.31)	<0.001 (*Χ*^2^ = 16.24)
**Severity level PHQ-9; *n* (%)**						
Minimal	87,490 (74.94)	80,989 (69.37)	<0.001 (*Χ*^2^ = 2243.03)	7,347 (39.44)	7,749 (41.45)	<0.001 (*Χ*^2^ = 41.20)
Mild	24,955 (21.37)	28,135 (24.10)		6,699 (35.83)	6,589 (35.24)	
Moderate	3,500 (3.00)	5,823 (4.99)		2,951 (15.78)	2,724 (14.57)	
Moderately severe	658 (0.56)	1,404 (1.20)		1,144 (6.12)	1,144 (6.12)	
Severe	147 (0.13)	399 (0.34)		527 (2.82)	489 (2.62)	
**Anxiety: GAD-7**						
*M* (*n*; SD)	2.80 (125,610; 2.83)	3.17 (125,610; 3.30)	<0.001 (*z* = −35.856)	5.96 (9,835; 4.52)	5.49 (9,835; 4.56)	<0.001 (*z* = 10.832)
Cutoff ≥10 [*n*, (%)]	4,077 (3.25)	6,279 (5.00)	<0.001 (*Χ*^2^ = 632.34)	1,943 (19.76)	1,648 (16.76)	< 0.001 (*Χ*^2^ = 46.46)
**Severity level GAD-7; *n* (%)**						
Minimal	99,427 (79.16)	91,405 (72.77)	<0.001 (*Χ*^2^ = 2450.18)	4,482 (45.57)	4,842 (49.23)	<0.001 (*Χ*^2^ = 80.10)
Mild	22,106 (17.60)	27.926 (22.23)		3,410 (34.67)	3,345 (34.01)	
Moderate	3,339 (2.66)	4,838 (3.85)		1,337 (13.59)	1,064 (10.82)	
Severe	738 (0.59)	1,441 (1.15)		606 (6.16)	548 (5.94)	

BL = Baseline assessment; FU = Follow-up assessment; ^a^comparison of scores and frequencies over time were based on Wilcoxon signed-rank tests, Asymptotic symmetry tests or McNemar tests (McNemar’s Χ^2^) for matched groups, as appropriate; PHQ-9 = 9-item Patient Health Questionnaire; severity levels of depression were defined as scores ranging from 0–4 minimal, 5–9 mild, 10–14 moderate, 15–19 moderately severe, and 20–27 severe; GAD-7 = 7-item Generalized Anxiety Disorder Scale; severity levels of anxiety are defined as scores ranging from 0–4: minimal anxiety, 5–9: mild anxiety, 10–14: moderate anxiety, 15–21: severe anxiety.

### Regression analyses—main effects model

In Table 3, the results of the main effects negative binomial regression model are displayed. Adjusting for covariates, severity of depressive symptoms was highest in younger participants [e.g., IRR_age 20 vs. age 45_ = 1.15 (95%-CI 1.09–1.20)] and declined with age, with the decline accelerating in those aged 60 years and older. Second, we found an effect for lifetime diagnosis of depression: having a self-reported psychiatric history nearly doubled the number of PHQ-9 symptoms (IRR = 1.91, 95%-CI 1.87–1.95), holding all other variables constant. Third, we found an overall increase in symptom severity of depression from before the pandemic to shortly after the first wave of the pandemic: regardless of age and psychiatric history and adjusted for covariates, the expected number of PHQ-9 symptoms increased by 12% (IRR = 1.12, 95%-CI 1.10–1.14) at follow-up compared to baseline assessment.

**Table 3:** Results of the main effect negative binomial regression model for estimating depression and anxiety severity.

	Depression			Anxiety		
	IRR	*p*-value	95% CI	IRR	*p*-value	95% CI
**Age**						
20	1.15	<0.001	[1.09–1.20]	0.99	0.585	[0.94–1.03]
30	1.07	<0.001	[1.04–1.10]	1.01	0.433	[0.99–1.04]
40	1.01	0.028	[1.00–1.03]	1.02	0.033	[1.00–1.03]
45	Ref.			Ref.		
50	0.97	<0.001	[0.96–0.98]	0.95	<0.001	[0.94–0.96]
60	0.87	<0.001	[0.85–0.89]	0.84	<0.001	[0.83–0.85]
70	0.58	<0.001	[0.56–0.59]	0.53	<0.001	[0.51–0.55]
	χ^2^ = 2895.27	<0.001		χ^2^ = 2876.51	<0.001	
**Time**						
Baseline	Ref.			Ref.		
FU	1.12	<0.001	[1.10–1.14]	1.15	<0.001	[1.12–1.17]
**Psychiatric history**						
No	Ref.			Ref.		
Yes	1.91	<0.001	[1.87–1.95]	1.92	<0.001	[1.89–1.96]

FU = Follow-up assessment; IRR = Incidence Rate Ratios; CI = confidence interval. Models were adjusted for gender, migration background, marital status, educational attainment, profession, and current employment.

Similar patterns for psychiatric history and time were found for anxiety. In addition, severity of anxiety symptoms steeply declined in older participants, but was in contrast to depression not higher in participants aged between 20 and 30 years.

### Regression analyses—interaction effects model

**Figure 1:**
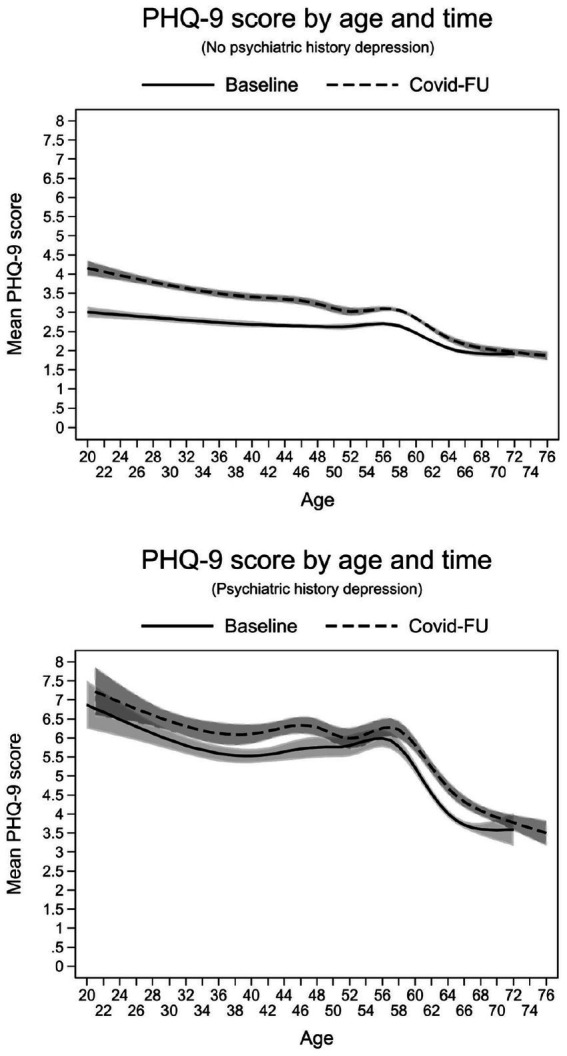
Interaction effects between age, time and psychiatric history of depression on the estimated severity of depressive symptoms.

In addition to the identified main effects, we also found significant interaction effects (Table 4). Regarding the severity of depressive symptoms, all three 2-way interactions between age, time and psychiatric history were found to be significant. Furthermore, a 3-way interaction between these risk factors was also evident, indicating that the severity of depression varied with age, and this variation was different for participants with and without a history of depression and changed from before to after the first wave of the pandemic. The complex relationship of age, time and psychiatric history with severity of depressive symptoms can be seen in Figure 1. First, participants with a depression history showed an overall higher estimated level of symptom severity compared to participants without depression history. At baseline, the mean PHQ-9 score was 3.44 (95%-CI 3.30–3.58) points higher in participants with a psychiatric history than in participants without a psychiatric history. At follow-up, the difference was smaller (2.81 points, 95%-CI 2.68–2.95), and this decrease in the group difference over time was statistically significant (Chi-square = 121.04, *p* < 0.001). Second, we found a general decreasing trend in PHQ-9 scores over the lifespan, although this was not similar at all ages and varied by psychiatric history. In people with a history of depression, levels peaked in their 20s and 30s, then plateaued until their late 50s and showed a sharp decline in older participants (left panel). Higher PHQ-9 levels in younger people and a converging trend in those over 60 were also found in the group without a history of depression (right panel). However, the estimated severity of symptoms among those younger than 50 years was remarkably higher during the pandemic than before, and this difference was more striking in the group without compared to the group with a history of depression.

**Table 4:** Results of the interaction effect negative binomial regression model for estimating depression and anxiety severity.

	Depression			Anxiety		
	IRR	*p*-value	95% CI	IRR	*p*-value	95% CI
**Age**						
20	1.18	<0.001	[1.12–1.24]	1.03	0.277	[0.98–1.07]
30	1.09	<0.001	[1.06–1.12]	1.03	0.019	[1.01–1.06]
40	1.02	0.043	[1.00–1.04]	1.02	0.015	[1.00–1.04]
45	Ref.			Ref.		
50	0.99	0.265	[0.98–1.01]	0.97	<0.001	[0.96–0.99]
60	0.91	<0.001	[0.89–0.93]	0.87	<0.001	[0.85–0.89]
70	0.67	<0.001	[0.64–0.70]	0.61	<0.001	[0.58–0.65]
	χ^2^ = 1385.77	<0.001		χ^2^ = 931.55	<0.001	
**Time**						
Baseline	Ref.			Ref.		
FU	1.45	<0.001	[1.31–1.60]	1.13	<0.001	[1.06–1.20]
**Psychiatric history**						
No	Ref.			Ref.		
Yes	2.69	<0.001	[2.13–3.40]	3.21	<0.001	[2.53–4.06]
**Interaction effects**						
Age × time	χ^2^ = 312.71	<0.001		χ^2^ = 240.71	<0.001	
Age × hist.	χ^2^ = 156.51	<0.001		χ^2^ = 48.78	<0.001	
Time × hist.	χ^2^ = 11.03	0.001		χ^2^ = 3.27	0.071	
Age × time × hist.	χ^2^ = 46.03	<0.001		χ^2^ = 31.28	<0.001	

FU = Follow-up assessment; IRR = Incidence Rate Ratios; CI = confidence interval; hist. = psychiatric history. Models were adjusted for gender, migration background, marital status, educational attainment, profession, and current employment.

**Figure 2:**
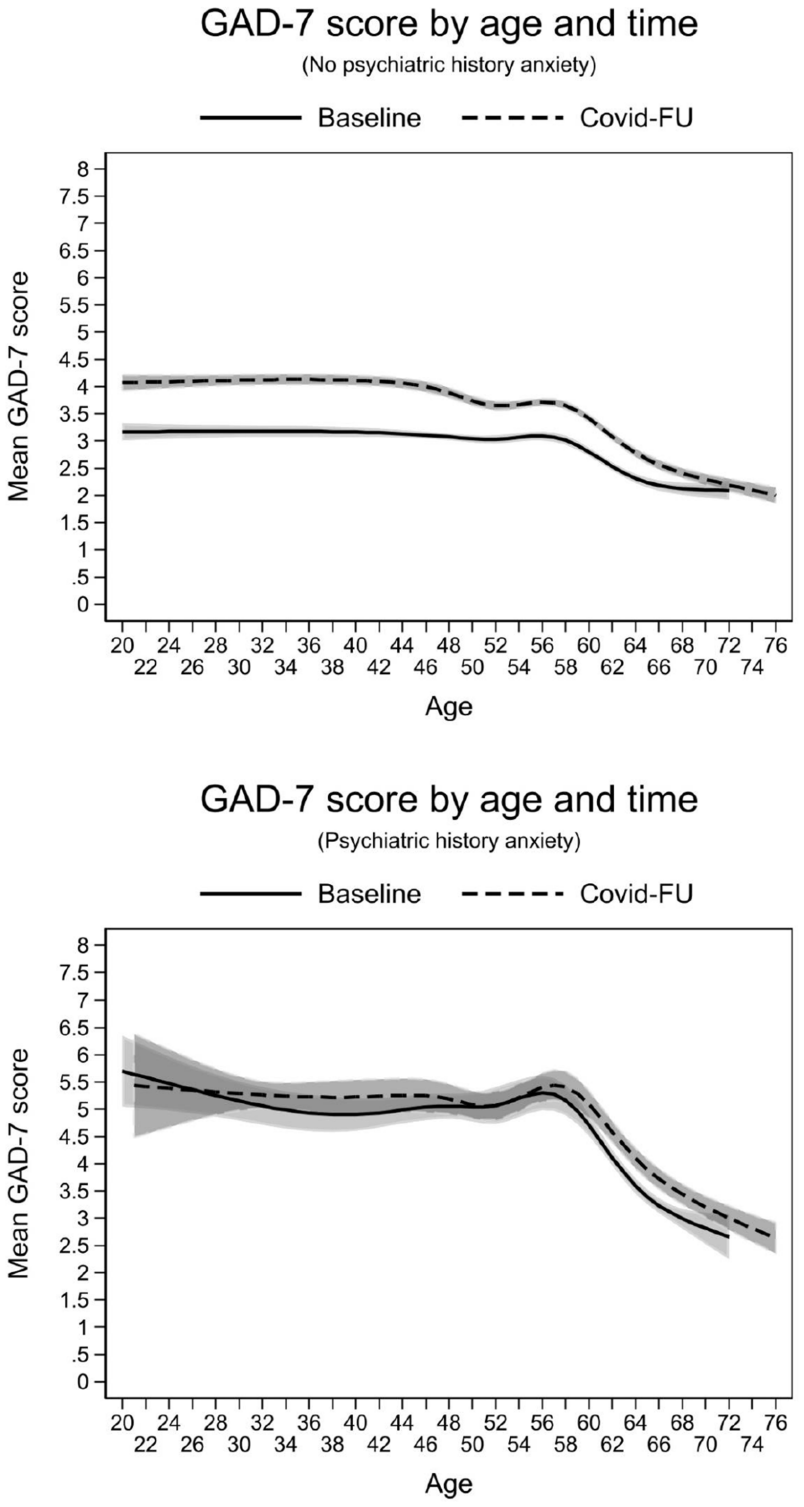
Interaction effects between age, time and psychiatric history of anxiety on the estimated severity of anxiety symptoms.

The results of the interaction effects model for anxiety were largely like those for depression, with one exception (Table 4). While the 2-way interaction contrasts for age X time and age X psychiatric history as well as the 3-way interaction age X time X psychiatric history similarly predicted severity of anxiety, no effect modification of time by self-reported history of anxiety emerged independent of age (χ^2^ = 3.27, *p* = 0.071). This implies that the average estimated increase in mean GAD-7 scores between the period before and during the pandemic across all ages (0.44 points, 95%-CI 0.36–0.51) was not different in both groups. Nevertheless, there were significant interactions with age (Figure 2). In participants under 50 years of age with no history of anxiety, symptom levels were consistently higher during the pandemic than before (right panel). This difference in mean GAD-7 scores over time, at an overall higher level, was not evident in the participants with a history of anxiety (left panel).

## Discussion

In the population-based NAKO study, participants with a psychiatric history of depression had on average a higher mean PHQ-9 score, i.e., more symptoms indicative of depression. In addition, mean PHQ-9 scores among people with/without depression were on average higher shortly after the first wave of the pandemic than before the pandemic. Regarding interaction effects, the results showed an increase in the mean PHQ-9 score from baseline to follow-up that was on average lower for those with a psychiatric history than for those without. Similar patterns were found for anxiety. Increased symptoms of depression and anxiety in the population as a short-term psychological consequence of the pandemic (first lockdown, initial) were observed—especially among younger people without a psychiatric history. Older people and people with a psychiatric history seemed more likely to be psychologically stable. Consequently, our study contradicts the findings that pre-existing symptoms of depression and anxiety are a vulnerability factor of depression and anxiety during the pandemic (27), especially among individuals with severe mental disorders (28).

For both depression and anxiety, we found clear associations with age, time and psychiatric history (main effects): in general, symptom severity decreased with age and was higher during the pandemic and higher for those with a psychiatric history of depression and anxiety. In line with the findings of Chen and colleagues (22), the current study adds more evidence to the nonlinear predictors of mental health disorders in the COVID-19 pandemic. More specifically, we observed similar interactions for depression and anxiety: higher scores in the pandemic were found among younger people and we observed a converging trend with age, particularly among those with no previous psychiatric history. In other words, in addition to those already burdened by their previous psychiatric history, younger people with no previous history had an increased risk of psychiatric disorders during the pandemic. Older adults, especially those with a psychiatric history remained stable during the pandemic according to our data. In line with the findings of a recent systematic review assessing the pandemic’s mental health impact on older adults independently of their (mental) health status (10), our findings underline changes in mental health symptoms during the early stages of the pandemic in older adults (≥60 years). Based on these findings, it seems that older adults are not at higher risk for negative mental health consequences caused by the pandemic. Previous perspectives argued that special circumstances during the pandemic such as decreased work load in some professions, less social engagement and consequently a reduced stress level might have had a relieving impact on mental stress, depression and anxiety levels (4). Parlapani et al. suggested that older age might serve as a protective factor against the adverse effects on mental health related to the COVID-19 pandemic, noting that older individuals are more likely to have experienced traumatic events, such as financial crises and natural disasters, both individually and collectively. Consequently, older individuals may be able to consider a global crisis such as the pandemic in a broader context, relativizing its impact and show higher resilience against negative COVID-19-related mental health impacts using adaptive resources (29). On the other hand, loneliness and social isolation was found to be an additional stressor with a potential negative impact on mental health, especially among young people. In the work of Lee and colleagues, younger people reacted to the first wave of the pandemic with increased depression, which could be almost entirely explained by increased feelings of loneliness (30). Recent data of the German National Cohort (NAKO) clearly demonstrated this assumed relationship: while overall loneliness increased during the pandemic, especially women and younger persons compared to men and older persons were affected. Also, higher levels of perceived loneliness were associated with higher levels of depression and anxiety. Persons reporting symptoms of depression and anxiety before the pandemic were more likely affected by stronger feelings of loneliness during the pandemic (15). Future studies should therefore examine in more detail the personal or health-related factors that support mental health and adaptation processes in older adults during challenging circumstances such as a pandemic.

In the present study, we observed a striking difference between depression and anxiety. While an increase of symptoms between before and during the pandemic was evident in both, and this increase was also age-sensitive for both disorders, only in depression this increase was found both age-dependent and age-independent, and group-specific (namely greater in the unaffected in general and the younger ones than in persons with psychiatric history and older ones). In the case of anxiety, this increase in unaffected participants over time was age-dependent, mainly observed in the younger unaffected in comparison to the younger affected participants. Overall, most of our findings underline the most recent meta-analytic and review findings on an international level showing a stable or even reduced mental health burden during the COVID-19 pandemic (3, 31).

So far, data for German-speaking countries are rare, so this study adds important findings to the existing evidence-base. Previous studies were mostly based on smaller samples of different age groups. For example, a longitudinal study examined a small sample of older individuals calling the psychiatric helpline during the first phase of the pandemic between April and June 2020 (*n* = 55, mean age 74.69 years). The authors found that individuals with a previous diagnosis of a psychiatric disease reported significantly higher levels of depressive and anxiety symptoms than those without a diagnosis (6). Further, Brosch et al. evaluated 1,268 participants (*n* = 622 healthy controls and *n* = 646 patients with major depression, bipolar disorder, schizophrenia or schizoaffective disorder) at baseline before (2014–2018) and during (April–May 2020) the first lockdown in Germany. They found that 30.5% of the patients reported worsened self-rated symptoms and a significantly higher subjective isolation since the pandemic (32). Another longitudinal original paper focused on older individuals (*n* = 32, mean age 77.94 years) with affective or anxiety disorders and reported no significant changes in psychopathology (33). Therefore, our current population-based study including individuals between 20 and 72 years makes an important contribution to the most recent international literature in the field. Further strengths of this study are the large sample across the German general population, covering a wide age range and allowing stratified analyses. Based on this, we were able to conduct detailed statistical analyses considering several factors such as age of participants and self-reported psychiatric history. The assessment of mental health was based on established and validated instruments (PHQ-9, GAD-7) for the dimensional recording of depression and anxiety symptoms. However, the dimensional assessment of psychiatric symptoms does not replace comprehensive psychiatric diagnostics.

Limitations refer to the design of the study. It would have been desirable to be able to investigate these effects beyond the first lockdown of the pandemic. However, results that are more recent suggest that the effects are also robust for later points in the pandemic. Accordingly, a recent review and meta-analysis showed that symptoms of anxiety and depression decreased over the course of the COVID-19 pandemic. This longitudinal study included at least two waves during the pandemic (34). In contrast to this, longitudinal data from a general population sample of 1,388 adults from Germany showed mixed results. The authors found that anxiety symptoms did not change from baseline to 12-month follow-up, while depressive symptoms and loneliness increased and life satisfaction decreased. Partly in harmony with our results, younger individuals or those with a history of mental disorders were found to be especially vulnerable to negative pandemic effects (35). Second, psychiatric symptoms were collected via self-reported measures that could have biased the results. On the other hand, data from a cohort study with several dimensional and categorical measures of depression showed that dimensional measures in the self-report even performed better than a diagnosis by the general practitioner (36).

Future research should expand on the long-term consequences of the pandemic, as such effects on mental health cannot be ruled out. In this context, special attention could be paid to the influences and consequences of Long-Covid (37). It would also be interesting to understand what consequences the pandemic has had for relatives of mentally ill people. Recent findings suggest that the COVID-19 pandemic was a burden for most people—particular for those with mental illness and their families (38). The aim of future studies should comprise the further investigation of specific burdens and coping strategies among relatives of people with mental illness. Also, the group of severely mentally ill people could be of particular interest, because it was shown that they were exposed to special risks (39, 40), including an increase in the risk for potential suicide (41). In conclusion, future efforts should focus on the improvement of mental health services facing global crisis and on the comprehension of underlying mechanisms and influences in medium-and long-term effects.

## Data Availability

The datasets analyzed during the current study are not publicly available due to privacy concerns but can be requested for via the NAKO transfer hub: https://transfer.nako.de/transfer/index.

## Group member of German National Cohort (NAKO) Consortium

NAKO e.V., Heidelberg, Germany
